# Compound C Protects Against Cisplatin-Induced Nephrotoxicity Through Pleiotropic Effects

**DOI:** 10.3389/fphys.2020.614244

**Published:** 2020-12-23

**Authors:** Fanghua Li, Anbang Sun, Genyang Cheng, Dong Liu, Jing Xiao, Zhanzheng Zhao, Zheng Dong

**Affiliations:** ^1^Department of Nephrology, The First Affiliated Hospital of Zhengzhou University, Zhengzhou, China; ^2^Department of Anatomy, Tongji Medical College, Huazhong University of Science and Technology, Wuhan, China; ^3^Department of Cellular Biology and Anatomy, Medical College of Georgia, Augusta University, Augusta, GA, United States

**Keywords:** compound C, cisplatin, acute kidney injury, p53, endoplasmic reticulum stress

## Abstract

AICAR (Acadesine/AICA riboside) as an activator of AMPK, can protect renal tubular cells from cisplatin induced apoptosis. But in our experiment, the dorsomorphin (compound C, an inhibitor of AMPK) also significantly reduced cisplatin induced renal tubular cells apoptosis. Accordingly, we tested whether compound C can protect cisplatin-induced nephrotoxicity and the specific mechanism. Here, we treated Boston University mouse proximal tubular cells (BUMPT-306) with cisplatin and/or different dosages of AICAR (Acadesine/AICA riboside) or compound C to confirm the effect of AICAR and compound C *in vitro*. The AMPK-siRNA treated cells to evaluate whether the protective effect of compound C was through inhibiting AMPK. Male C57BL/6 mice were used to verify the effect of compound C *in vivo*. Both compound C and AICAR can reduce renal tubular cells apoptosis in dose-dependent manners, and compound C decreased serum creatinine and renal tubular injury induced by cisplatin. Mechanistically, compound C inhibited P53, CHOP and p-IREα during cisplatin treatment. Our results demonstrated that compound C inhibited AMPK, but the renal protective effects of compound C were not through AMPK. Instead, compound C protected cisplatin nephrotoxicity by inhibiting P53 and endoplasmic reticulum (ER) stress. Therefore, compound C may protect against cisplatin-induced nephrotoxicity through pleiotropic effects.

## Introduction

Acute kidney injury (AKI) characterized by the rapid decline of renal function is often caused by the renal ischemia-reperfusion, sepsis, and nephrotoxins such as cisplatin, vancomycin, and gentamicin. The pathogenesis of AKI is complicated, including several cell types, cellular processes, and molecular regulators and mediators. The major pathological feature of AKI is sublethal even lethal damage of the renal tubular epithelial cells (Yang et al., 2010). In addition to the association with high morbidity and mortality, AKI is a key risk factor for the development of chronic kidney disease (CKD) and end-stage renal disease (ESRD) (Ishani et al., 2009; Venkatachalam et al., 2010).

Cisplatin is used as a first-line chemotherapeutic agent for cancer. But cisplatin is also well-known for its toxicity in normal tissues and organs, especially the kidneys. The nephrotoxicity constraints its clinical application. The pathogenesis of cisplatin nephrotoxicity involves a variety of factors and signaling pathways. In this condition, the DNA damage response, endoplasmic reticulum (ER) stress, mitochondrial damage, and other stress responses in renal tubular cells, leading to renal tubular damages and renal dysfunction. P53, a well-known tumor suppression protein, has been clarified to play an important role in the pathogenesis of cisplatin-induced nephrotoxicity or AKI (Jiang et al., 2006; Jiang and Dong, 2008). P53 is evidently activated in renal proximal tubular cells during cisplatin treatment, and its activation can lead to the tubular cell death through transactivation of the proapoptotic gene, including P53 up-regulated modulator of apoptosis (PUMA-α) and P53-induced protein with a death domain (PIDD) (Seth et al., 2005; Pabla and Dong, 2008; Yan et al., 2016). As a result, pharmacological or genetic inhibition of P53 is beneficial to cisplatin nephrotoxicity (Seth et al., 2005; Jiang et al., 2006).

AMP-activated protein kinase (AMPK) is an energy sensor that regulates energy metabolism, cell growth, proliferation, and apoptosis. AMPK inhibits biosynthetic pathways through acetyl CoA carboxylase and mTOR to maintain essential energy and nutrients in times of metabolic crisis. Compound C (also called dorsomorphin), commonly used as the inhibitor of AMPK. In recent reports especially in the kidney, the biochemical and cellular effects of compound C have been attributed to its inhibitory action toward AMPK (Wei et al., 2015; Wang et al., 2018).

In this study, we observed that AMPK was activated in renal tubular cells upon cisplatin treatment. Compound C inhibited AMPK activity and apoptosis of renal tubular cells induced by cisplatin. However, it appeared that the anti-apoptotic effects of this compound were AMPK independent. Instead, compound C protected cisplatin nephrotoxicity by inhibiting P53 and ER stress. Together, these results support the therapeutic potential of compound C in cisplatin-induced AKI.

## Materials and Methods

### Materials

Male C57BL/6 mice (8–10 weeks) were purchased from the experimental animal center of Zhengzhou University (Zhengzhou, Henan). The BUMPT cell line (Boston University mouse proximal tubular cell, BUMPT-306) was originally from Drs. John Shwartz William and Lieberthal at Boston University (Bhatt et al., 2010). The primary antibodies such as anti-P53, anti-AMPK, anti-phospho-AMPK (p-AMPK), anti-cleaved PARP (c-PARP), anti–cleaved caspase-3 (c-caspase3), and anti-CHOP antibodies were from Cell Signaling Technology (1:1,000, Danvers, MA, United States); anti–phospho-(serine-15)-P53 (p-P53), and anti-phospho-(s724)-IRE1α (p-IRE1α) antibody were from Abcam (1:1,000, Cambridge, United Kingdom); anti–β-actin(1:1,000) was from Sigma-Aldrich (St. Louis, MO, United States); all secondary antibodies used for immunoblot analysis were purchased from Thermo Fisher Scientific (1:5,000, Waltham, MA, United States). Compound C and AICAR were purchased from Abcam, and cisplatin was from Sigma-Aldrich.

### Mouse Model of Cisplatin Nephrotoxicity

Male C57BL/6 mice were injected intraperitoneally with cisplatin (30 mg/kg) oncely. The control group of mice were injected with the same dose of saline. To study the effects of compound C, compound C was dissolved in DMSO and injected intraperitoneally at 10 mg/kg body weight 1 h before the injection of cisplatin. The no-compound C animals were administered with a comparable volume of DMSO. All the mice were euthanized at 72 h. We performed the animal experiments according to the protocol approved by the Care and Use of Laboratory Animals Institutional Committee of the first affiliated hospital of Zhengzhou University.

### Cells and Cisplatin Treatment

BUMPT cells were cultured in DMEM/F12 medium containing 10% fetal bovine serum and 10% streptomycin. Lipo2000 (Invitrogen, Carlsbad, CA, United States) was used for transfection, and the sequences of AMPK siRNA were KD1 (sense: GGAGAGCUAUUUGAUUAUATT, antisense: UAUAA UCAAAUAGCUCUCCTT), KD2 (sense: GGGAACACGAGU GGUUUAATT, antisense: UUAAACCACUCGUGUUCCCTT), and scramble as negative control (sense: UUCUCCGAA CGUGUCACGUTT, antisense: ACGUGACACGUUCGGAG AATT). The immunoblot was used to evaluate the knockdown efficiency. The transfected cells were cultured with 20 μM cisplatin, which induced obvious apoptosis as previously indicated (Bhatt et al., 2010).

### Examination of Apoptosis

Apoptosis in cell cultures was analyzed according to the cell morphology. In brief, cells were stained with Hoechst33342 (Beyotime Biotechnology, Jiangsu, China) and monitored by phase-contrast microscopy. Typical apoptotic morphology including cellular and nuclear condensation and fragmentation were counted to determine the percentage of apoptosis. TUNEL assay was used to detect apoptosis of renal tissues. In short, the paraffin sections of kidneys were stained according to the instructions of *in situ* cell death detection kit from Roche diagnosis company (Roche, Basilea, Switzerland).

### Analysis of Renal Function and Histology

Renal function was measured by serum creatinine and blood urea nitrogen (BUN) according to the manufacturer’s instructions of commercial kits from the Bioassay System (Hayward, CA, United States). In renal histology, 4% paraformaldehyde was used to fix renal tissue and embedded in paraffin. The paraffined samples were sectioned for hematoxylin-eosin (HE), immunohistochemistry staining and TUNEL assay. The renal tubular injury was scored in a blinded manner by the percentage of injured tubules with loss of brush border, cell lysis, and cast formation: 0, no damage; 1, <25%; 2, 25–50%; 3, 50–75%; 4, >75%.

### Immunoblot Analysis

The buffer containing 2% SDS and protease inhibitor cocktail was used to lysis the cells and kidney tissues to extract proteins. Equal amounts of protein were loaded in reducing gel electrophoresis and transferred according to standard procedures. 5% bovine serum albumin was used to block the blots, and then the blots were exposed to specific primary antibodies overnight at 4°C. Finally, the blots membranes were incubated with the horseradish peroxidase-conjugated secondary antibodies, and the blots signal were revealed with an ECL Kit (EMD Millipore, Billerica, MA, United States).

### Immunohistochemistry

Immunohistochemical staining for p-P53 was performed according to the protocol. Briefly, deparaffinized sections were treated with 0.1 M sodium citrate (pH 6.0) at 95–100°C for 20 min for antigen retrieval and sequentially incubated with 3% H_2_O_2_, blocking buffer (2% normal goat serum), anti-p-P53 (1:100) at 4°C overnight and biotinylated goat anti-rabbit secondary antibody for 20 min at room temperature. Antibody diluent treated the negative controls. The color was developed with a DAB kit. Quantification of p-P53-positive nuclear staining cells were evaluated by counting about 20 fields (original magnification, ×200) randomly selected from each section.

### Statistical Analyses

The quantitative data reported in this study are representative of at least 3 separate experiments and shown as means ± SD. Statistical analysis was conducted by the GraphPad Prism 5 software (GraphPad Software, Inc., La Jolla, CA, United States). Statistical differences among multiple groups were determined by One-way ANOVA followed by Tukey’s multiple comparison test. Significant difference was considered when a value of *P* < 0.05.

## Results

### Compound C Reduces BUMPT Cells Apoptosis Induced by Cisplatin

**FIGURE 1:**
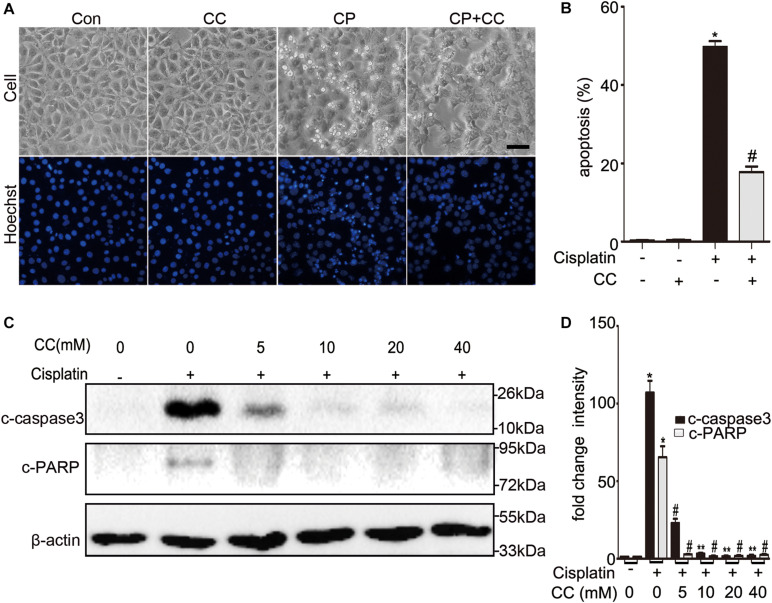
Compound C reduces cisplatin-induced apoptosis in BUMPT cells. **(A)** Morphology. BUMPT cells were incubated in 20 μM cisplatin with or without compound C (CC) for 24 h and then stained with Hoechst 33342. Cellular and nuclear morphology were recorded by phase-contrast and fluorescence microscopy (original magnification, ×200). Scale bar, 50 μm. **(B)** Apoptosis percentage. About 200 cells in each group were evaluated to determine the percentage of cells with typical apoptotic morphology. **(C,D)** BUMPT cells were treated with or without cisplatin plus different concentrations of CC (0, 5, 10, 20, and 40 mM) for 24 h. Whole cell lysates were collected for immunoblot analysis of c-caspase3, c-PARP, and β-actin as loading control. **(C)** Representative immunoblots. **(D)** Densitometry of c-caspase3 and c-PARP signals. The c-caspase3 signals were normalized to the β-actin signal of the same samples to determine the ratios. The ratios of control group (in the absence of cisplatin and CC) were arbitrarily set as 1. Means ± SD (*n* = 3). *P < 0.001 vs. cisplatin-only-treated cells, ^#^*P* < 0.001 vs. cisplatin-only-treated cells, and **P < 0.01 vs. cisplatin + 5 mM CC treated cells.

We initially examined whether the addition of compound C was protective of cisplatin nephrotoxicity in cell culture. BUMPT cells were cultured in 20 μM of cisplatin in the presence or absence of 20 mM compound C for 24 h. To evaluate the renal tubular cells apoptosis, we used morphologic assay and immunoblot to analyze the cleaved caspase3 and PARP (c-caspase3 and c-PARP). As shown in Figure 1A, compared with the cells treated with cisplatin only, there were fewer apoptosis cells in the group treated with cisplatin plus compound C. Quantification by counting showed that cisplatin induced ∼50% apoptosis, while compound C reduced the apoptosis to nearly 20% (shown in Figure 1B). Consistent with the results of morphology, compound C decreased the expression of c-caspase3 and c-PARP in cisplatin treatment, and the protective effect of compound C was dose-dependent (shown in Figures 1C,D).

### Compound C Attenuates Cisplatin-Induced Nephrotoxicity in Mice

**FIGURE 2:**
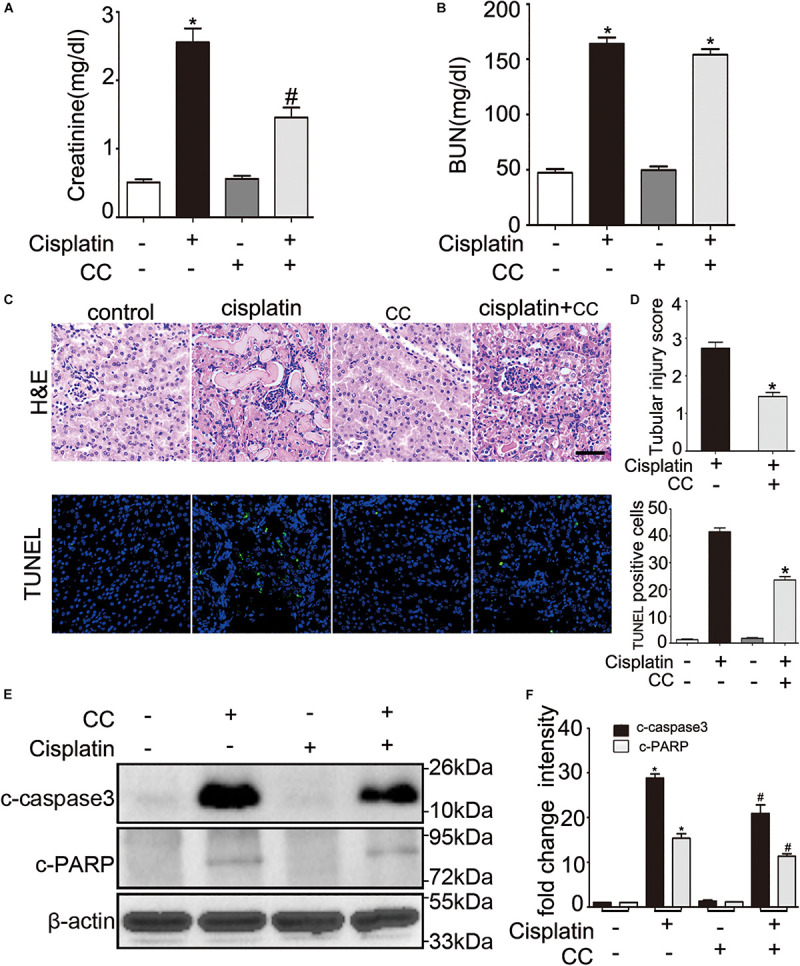
Compound C attenuates cisplatin induced AKI in mice. C57BL/6 mice (male, 8–10 weeks) were administered cisplatin (30 mg/kg) with CC (10 mg/kg) or with saline as control. Mice were euthanized at 72 h. **(A,B)** Blood samples were collected for measurements of serum creatinine **(A)** and BUN **(B)**. **(C–F)** Renal tissues were collected for histologic analysis and immunoblot. **(C)** Upper panel: Representative histology of kidney cortex by hematoxylin and eosin (H&E) staining. Lower panel: Representative images of TUNEL staining of kidney tissues. Scale bar, 200 μm. **(D)** Upper panel: Histopathological score of tubular damage. Lower panel: Quantification of TUNEL-positive cells/mm^2^ in kidney tissues. **(E)** Immunoblot analysis of c-caspase3, c-PARP and β-actin as protein loading control. **(F)** Densitometry of c-caspase3 and c-PARP. Means ± SD (*n* = 4). *P < 0.001 vs. the control groups, ^#^*P* < 0.05 vs. cisplatin-only-treated groups.

We then tested the renoprotective effect of compound C during cisplatin-induced nephrotoxicity in mice. C57BL/6 mice were intraperitoneally injected with compound C (10 mg/kg) or the same dose of DMSO solution 1 h before cisplatin (30 mg/kg) was administered, and mice were sacrificed after 72 h. As shown in Figure 2A, mice treated with cisplatin plus compound C had a notably lower level of serum creatinine than those treated with cisplatin only (Figure 2B). In line with the serum creatinine, there was less severe renal tubular damage in mice treated with cisplatin plus compound C in comparison to the mice treated with cisplatin alone (shown in Figures 2C,D, upper panel). Also, the TUNEL assay revealed that compound C reduced the apoptosis of renal tubular cells in mice (shown in Figures 2C,D, lower panel). Furthermore, the immunoblot analysis demonstrated that compound C alleviated c-caspase 3 and c-PARP induced by cisplatin in kidney tissues (shown in Figures 2E,F). In general, these *in vivo* experiments provided compelling evidences for the protective role of compound C in cisplatin-induced nephrotoxicity.

### Compound C Inhibits Cisplatin-Induced Activation Not Through AMPK

**FIGURE 3:**
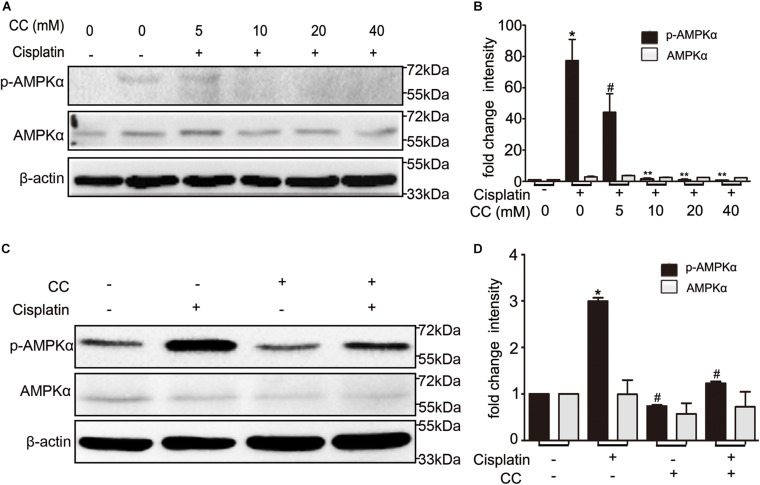
Compound C inhibits AMPK during cisplatin treatment. **(A,B)** BUMPT cells were treated with cisplatin plus different concentrations of CC (0, 5, 10, 20, and 40 mM) for 24 h. Whole cell lysates were collected for immunoblot analysis of AMPK, p-AMPK, and β-actin as loading control. **(C,D)** C57BL/6 mice (male, 8–10 weeks) were administered cisplatin (30 mg/kg) with CC (10 mg/kg) or with DMSO as control. Mice were euthanized at 72 h. Renal tissues were collected for immunoblot analysis of AMPK, p-AMPK, and β-actin as loading control. **(A,C)** Representative immunoblots. **(B,D)** Densitometry of AMPK and p-AMPK signals. *P < 0.001 vs. the control groups, ^#^*P* < 0.01 vs. cisplatin-only-treated groups, **P < 0.01 vs. cisplatin + 5 mM CC treated cells.

**FIGURE 4:**
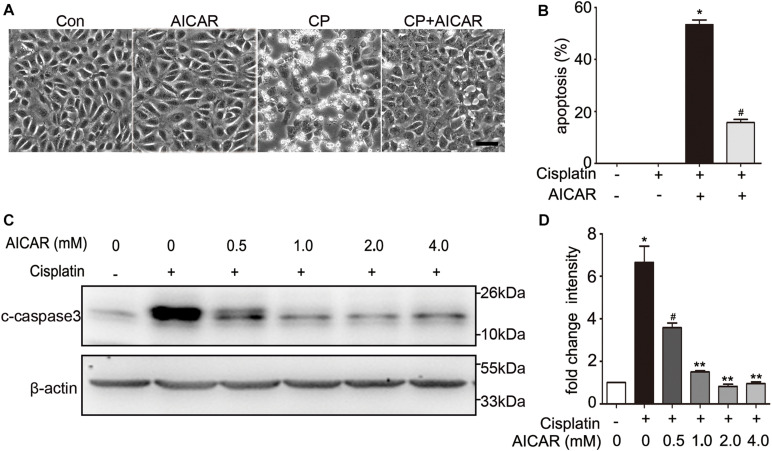
The effect of AICAR on cisplatin induced apoptosis in BUMPT cells. **(A)** Representative morphology. BUMPT cells were incubated in the presence or absence of cisplatin (20 μM) with or without AICAR for 24 h. Cellular morphologies were recorded by microscopy (original magnification, ×200). Scale bar, 50 μm. **(B)** Apoptosis percentage. About 200 cells in each group were evaluated to determine the percentage of cells with typical apoptotic morphology. **(C,D)** BUMPT cells were treated in presence or absence of cisplatin with different concentrations of AICAR (0, 0.5, 1.0, 2.0 and 4.0 mM) for 24 h. Whole cell lysates were collected for immunoblot analysis of c-caspase3, and β-actin as loading control. **(C)** Representative immunoblots. **(D)** Densitometry of c-caspase3 signals. Means ± SD (*n* = 3). *P < 0.001 vs. the control groups, ^#^*P* < 0.01vs. cisplatin-only-treated cells, and **P < 0.01 vs. cisplatin + 0.5 mM AICAR treated cells.

**FIGURE 5:**
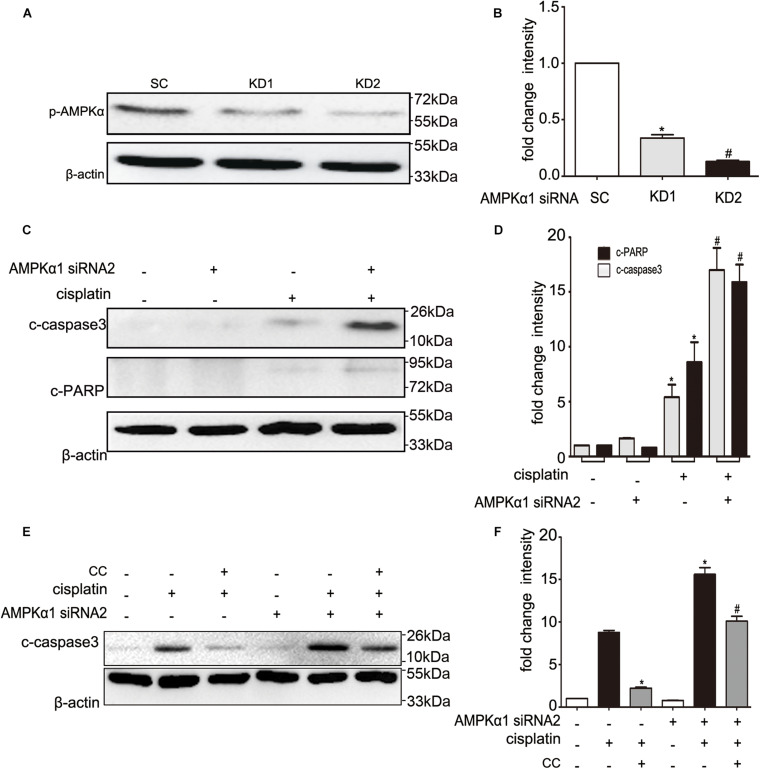
Effects of AMPK siRNA on cisplatin and compound C treated BUMPT cells. **(A,B)** BUMPT cells were transfected with 50 nM scrambled sequence (SC) or AMPK siRNA (KD1, KD2). The knock down efficiency was determined by immunoblot analysis of p-AMPK and β-actin (loading control). **(A)** Representative immunoblots. **(B)** Densitometry of p-AMPK signals. Means ± SD (*n* = 3). *P < 0.001 vs. SC cells, ^#^*P* < 0.05 vs. KD1 cells. **(C,D)** The SC or KD2 cells were then treated in presence or absence of cisplatin (20 μM) for 24 h. Whole cell lysates were collected for immunoblot analysis of c-caspase3, c-PARP, and β-actin as loading control. **(C)** Representative immunoblots. **(D)** Densitometry of c-caspase3 and c-PARP signals. Means ± SD (*n* = 3). *P < 0.01 vs. untreated SC cells, and ^#^*P* < 0.05 vs. cisplatin treated SC cells. **(E,F)** The SC or KD2 cells were then treated in presence or absence of cisplatin (20 μM) or CC (20 mM) for 24 h. Whole cell lysates were collected for immunoblot analysis of c-caspase3, and β-actin as loading control. **(E)** Representative immunoblots. **(F)** Densitometry of c-caspase3 signals. Means ± SD (*n* = 3). *P < 0.001 vs. cisplatin-only treated SC cells, and ^#^*P* < 0.01 vs. cisplatin-only treated KD2 cells.

To identify whether the protective effect of compound C is through inhibition of AMPK, we first detected the expression of total and phosphorylated AMPK during cisplatin treatment with or without compound C. As shown in Figure 3, the phosphorylated AMPK (p-AMPK) were increased in cisplatin treated BUMPT cells and mice kidneys, while compound C reduced the phosphorylation of AMPK in a concentration dependent manner. Then we used AICAR (an agonist of AMPK) to test the effect of AMPK in cisplatin induced nephrotoxicity. Unexpectedly, when compared to the cisplatin treated BUMPT cells, apoptosis was also significantly attenuated in cisplatin cotreated with AICAR group (shown in Figures 4A,B). This result was verified by the immunoblot of c-caspase3, which was reduced by AICAR in a dose-dependent manner (shown in Figures 4C,D). We further verified this result by genetic means of reducing AMPK. The catalytic α1 subunit of AMPK plays an important role in the biological activity of AMPK. Indeed siRNA-mediated knockdown of the α1 subunit reduced 80–90% of phosphorylated AMPK levels (shown in Figures 5A,B). To address the endogenous AMPK is required for the protection of cisplatin-induced nephrotoxicity, we treated scramble control (SC) and AMPKα1 siRNA (KD2) BUMPT cells with 20 μM cisplatin. Interestingly, AMPK-silenced BUMPT cells showed more apoptosis when compared to SC group (shown in Figures 5C,D). We also compared the effect of compound C on cisplatin treated SC and KD2 BUMPT cells, as shown in Figures 5E,F, the KD2 cells showed more apoptosis which can be attenuated by compound C. Collectively, these results suggest that AMPK plays a protective role in cisplatin-induced nephrotoxicity, and the renal protective effect of compound C was not through the inhibition of AMPK.

### Compound C Suppresses P53 Activation in Cisplatin Nephrotoxicity

**FIGURE 6:**
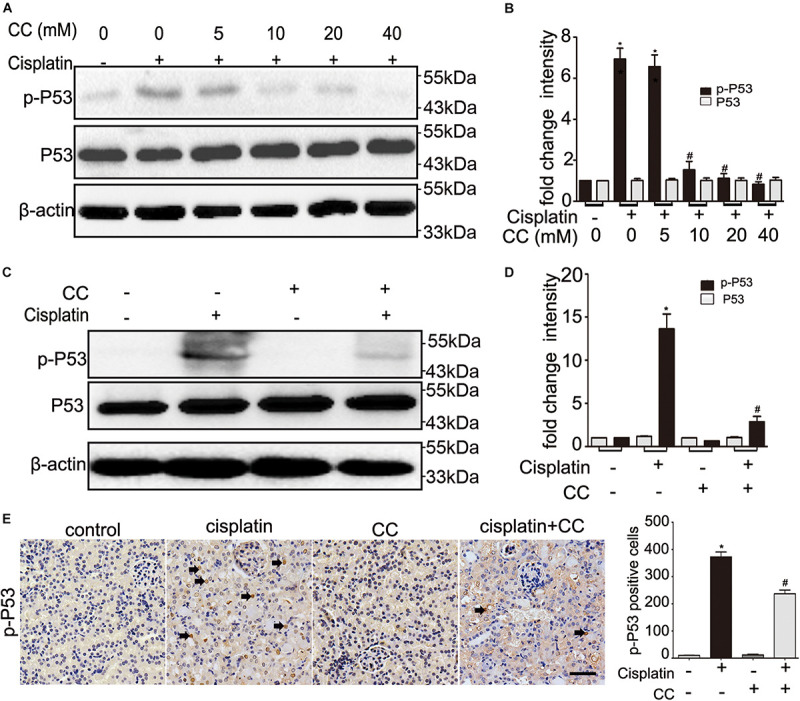
Compound C attenuates cisplatin nephrotoxicity by suppressing P53. **(A,B)** BUMPT cells were treated with cisplatin (20 μM) plus different concentrations of CC (0, 5, 10, 20, and 40 mM) for 24 h. Whole cell lysates were collected for immunoblot analysis of p-P53, P53, and β-actin (loading control). **(C–E)** C57BL/6 mice (male, 8–10 weeks) were administered cisplatin (30 mg/kg) with CC (10 mg/kg) or with saline as control. Mice were euthanized at 72 h. Renal tissues were collected for immunoblot analysis and immunohistochemical of p-P53. **(A,C)** Representative immunoblots. **(B,D)** Densitometry of p-P53 or P53 signals. **(E)** Left panel: Representative immunohistochemistry. Arrows were used to indicate a few positive nucleis. Scale bar, 200 μm. Right panel: quantification of p-P53 positive cells/mm^2^. *P < 0.0001 vs. the control groups, and ^#^*P* < 0.01 vs. cisplatin-only-treated groups.

As our above results indicate that the protective effect of compound C is not dependent on AMPK, we further examined the effect of compound C on P53, which plays an important role in cisplatin induced-nephrotoxicity. We cultured BUMPT cells in 20 μM cisplatin in the presence of different concentrations of compound C for 24 h and tested the expression of P53 by immunoblot analysis. Compared with cisplatin only treated group, the phosphorylated P53 (p-P53) levels were increased in cisplatin-treated cells, and importantly, compound C attenuated p-P53 in a dose-dependent manner (shown in Figure 6A). This suppressive effect of compound C on p-P53 was further supported by quantification of densitometry (shown in Figure 6B). *In vivo*, cisplatin increased P53 phosphorylation in kidney tissues of mice, while compound C reduced the phosphorylation of P53 during cisplatin treatment (shown in Figure 6C). Densitometry showed that p-P53 was dramatically increased by cisplatin, which was attenuated by compound C (shown in Figure 6D). Furthermore, when compared with cisplatin-only treated group, the immunohistochemical (IHC) staining showed the p-P53 positive cells were less than cisplatin plus compound C treated group (shown in Figure 6E). Taken together, these results indicated that compound C may protect the kidneys against cisplatin by suppressing the activation of P53.

### Compound C Suppresses ER Stress in Cisplatin Nephrotoxicity

**FIGURE 7:**
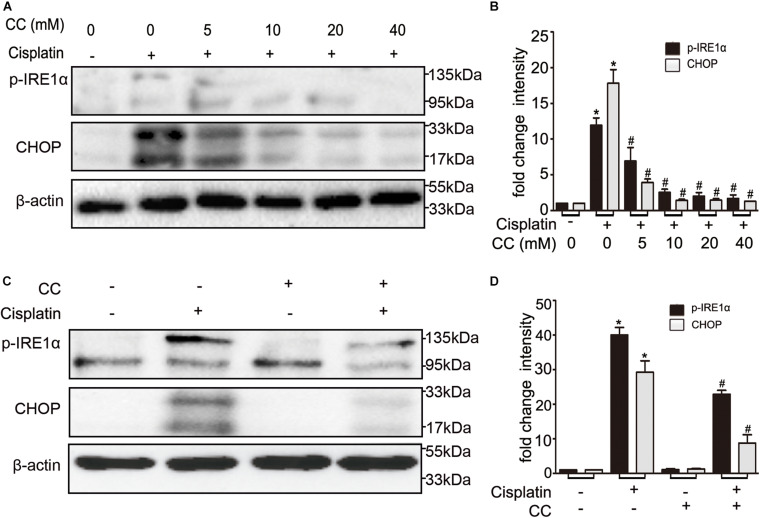
Compound C inhibits cisplatin nephrotoxicity by suppressing ER stress. **(A,B)** BUMPT cells were treated with cisplatin (20 μM) plus different concentrations of CC (0, 5, 10, 20, and 40 mM) for 24 h. Whole cell lysates were collected for immunoblot analysis of p-IRE1α, CHOP and β-actin (loading control). **(C,D)** C57BL/6 mice (male, 8–10 weeks) were administered cisplatin (30 mg/kg) with CC (10 mg/kg) or with saline as control. Mice were euthanized at 72 h. Renal tissues were collected for immunoblot analysis of p-IRE1α, CHOP and β-actin (loading control). **(A,C)** Representative immunoblots. **(B,D)** Densitometry of p-IRE1α and CHOP signals. *P < 0.0001 vs. the control groups, and *^#^P* < 0.01 vs. cisplatin-only-treated groups.

We further examined the effect of compound C on ER stress, which was shown to mediate cisplatin induced AKI (Yan et al., 2018). When compared with control cells, the ER stress markers such as p-IRE1α and CHOP were increased in cisplatin-treated cells, which were attenuated by compound C in a dose-dependent manner (shown in Figure 7A). This suppressive effect was further supported by quantification of densitometry (shown in Figure 7B). We also determined this phenomenon *in vivo*, as shown in Figure 7C, cisplatin induced the expression of p-IRE1α and CHOP in kidney tissues in mice, while compound C suppressed p-IRE1α and CHOP during cisplatin treatment. Densitometry analysis indicated that compound C attenuated ER stress markers induced by cisplatin (shown in Figure 7D). Collectively, these results suggested that compound C may protect kidneys from cisplatin nephrotoxicity by suppressing ER stress.

## Discussion and Conclusion

There is still lack of effective treatment for cisplatin-induced nephrotoxicity. In this study, we have shown that compound C reduces renal tubular cells apoptosis and maintains the renal function during cisplatin-induced nephrotoxicity. Mechanistically, compound C suppresses P53 and ER stress in cell and mouse models of cisplatin nephrotoxicity, contributing to a protective effect on AKI.

Compound C, a pharmacological inhibitor of AMPK, suppressed the activation of AMPK *in vitro* and *in vivo* in the present study, but it seems that the protective effect of compound C was not through inhibiting AMPK. In our study, we used the AMPK activator AICAR to test the effect of AMPK during cisplatin treatment. Interestingly, we found that AICAR also reduced cisplatin induced apoptosis (shown in Figure 4). We further verified this result by knocking down AMPK with siRNA (shown in Figure 5). AMPK silencing increased BUMPT cells apoptosis. These findings are inconsistent with other study, in which compound C was shown to increase renal tubular cells apoptosis in cisplatin nephrotoxicity (Wei et al., 2015).

The role of AMPK in AKI is perplexing. Some studies showed that AMPK protects against ischemic AKI and uric acid-induced renal injury by reducing the apoptosis of renal tubular epithelial cells (Lieberthal et al., 2011; Kim et al., 2015; Wei et al., 2015; Tsogbadrakh et al., 2019; Xiao et al., 2019), while others argued that AMPK can aggravate AKI and promote renal fibrosis (Taub and Cutuli, 2012; Wang et al., 2018; Jin et al., 2020). Due to its effect on increasing insulin sensitivity, more efforts are paid on developing activators of AMPK. Unfortunately, the compound C is the only inhibitor of AMPK that has been used to study the biological role of AMPK. It was suggested that compound C was an efficient anti-glioma agent that the anti-tumor effect was AMPK independent in recent studies (Liu et al., 2014; Wei et al., 2015). Actually, there are reports that compound C may inhibit other protein kinases more effectively than AMPK (Bain et al., 2007; Vogt et al., 2011).

In the present study, we showed that compound C reduced the apoptosis of renal tubular cells and maintained renal function during cisplatin treatment in cells and mice. Moreover, we demonstrated that the renal protective effect of compound C may not be through the inhibition of AMPK. We speculate that this discrepancy may be due to the pleiotropic role of compound C. Actually, compound C inhibited the activation of P53. Also compound C suppressed ER stress during cisplatin nephrotoxicity, as indicated by the suppression of p-IRE1α and CHOP.

It has been demonstrated that P53 was a major factor in AKI by mediating tubular cell apoptosis (Ying et al., 2014; Chen et al., 2016b; Tang et al., 2019). Previous study indicated that activated AMPK suppresses P53 (Baek et al., 2020), other research demonstrated that compound C suppresses P53 activation through inhibition of AMPK (Jin et al., 2020), while another study suggested that compound C could induce P53 expression, phosphorylation and nuclear translocation of the p53 in cancers (Huang et al., 2013; Zhao et al., 2018). The current study has provided *in vitro* and *in vivo* evidences for the suppressive effect of compound C on P53. When BUMPT cells subjected to cisplatin treatment, compound C reduced the activation of P53 in a concentration-dependent manner (shown in Figure 6A). Consistently, *in vivo* studies also verified that compound C reduced the phosphorylation of P53 during cisplatin treatment (shown in Figure 6). Along with the reduction of p-P53 levels by compound C, renal tubular cells apoptosis was reduced. These findings suggested that compound C suppresses P53-mediated tubular cell apoptosis during cisplatin-induced nephrotoxicity. But how compound C regulates P53 remains unclear and it will be an interesting project for future research.

Endoplasmic reticulum (ER) stress is also called the unfolded protein response (UPR), which is characterized by the accumulation of unfolded or misfolded proteins in the ER lumen. Studies showed that ER stress markers such as XBP1 and GRP78 were elevated in cisplatin-treated rats (Peyrou et al., 2007). Moreover, the elevated ER stress was associated with severe renal injury during cisplatin treatment (Long et al., 2017). Further studies suggested that ER stress contributed to renal tubular cells apoptosis induced by cisplatin, while suppressing ER stress can reduce tubular cells apoptosis (Chen et al., 2016a). Some other studies demonstrated that the activation of AMPK inhibits ER stress and ameliorates renal fibrosis and apoptosis (Lee et al., 2012; Kim et al., 2015; Huang et al., 2020), while other study indicated that inhibition of AMPK could diminish ER stress to prevent gastric cancer progression (Sun et al., 2019). In our study, we determined that the ER stress markers IRE-1α and CHOP were induced by cisplatin both in BUMPT cells and in mice kidneys (shown in Figure 7). Interestingly, compound C reduced the expression of p-IRE1α and CHOP during cisplatin treatment (shown in Figure 7). In line with our study, compound C was verified to attenuate the ER stress related proteins such as ATF4, PERK and GRP78 in glucose deprived human fibrosarcoma cells (Saito et al., 2012; Liu et al., 2014).

In the research of renal protective strategy against cisplatin-induced nephrotoxicity, it is important to consider the chemotherapy effect in cancers. An ideal renal protective agent should protect kidneys without diminishing the anticancer efficacy of cisplatin. In fact, many researches showed that compound C was an efficient antineoplastic agent, which promoted the apoptosis of human breast, liver, lung, prostate, colorectal, skin, and cholangiocarcinoma cancer cells, as well as multiple myeloma cells (Baumann et al., 2007; Jin et al., 2007, 2009; Park et al., 2009; Yang et al., 2012; Dai et al., 2013; Huang et al., 2013). Our findings demonstrate that compound C alleviates cisplatin induced nephrotoxicity. Thus, compound C may be an ideal strategy for renal protection during cisplatin chemotherapy in cancer patients because it may protect kidneys while promoting the chemotherapy effect.

## Data Availability Statement

The raw data supporting the conclusions of this article will be made available by the authors, without undue reservation, to any qualified researcher.

## Ethics Statement

The animal study was reviewed and approved by the Care and Use of Laboratory Animals Institutional Committee of the first affiliated hospital of Zhengzhou University.

## Author Contributions

FL and ZZ designed the research. ZD and JX revised the manuscript. AS performed the experiments. GC and DL analyzed the data. FL drafted the manuscript. All authors contributed to the article and approved the submitted version.

## Conflict of Interest

The authors declare that the research was conducted in the absence of any commercial or financial relationships that could be construed as a potential conflict of interest.
